# Herbal Extracts That Reduce Ocular Oxidative Stress May Enhance Attentive Performance in Humans

**DOI:** 10.1155/2016/4292145

**Published:** 2016-12-20

**Authors:** Hohyun Cho, Moonyoung Kwon, Hyojung Jang, Jee-Bum Lee, Kyung Chul Yoon, Sung Chan Jun

**Affiliations:** ^1^School of Information and Communications, Gwangju Institute of Science and Technology, Gwangju, Republic of Korea; ^2^Department of Dermatology, Chonnam National University Medical School and Hospital, Gwangju, Republic of Korea; ^3^Department of Ophthalmology, Chonnam National University Medical School and Hospital, Gwangju, Republic of Korea

## Abstract

We used herbal extracts in this study to investigate the effects of blue-light-induced oxidative stress on subjects' attentive performance, which is also associated with work performance. We employed an attention network test (ANT) to measure the subjects' work performance indirectly and used herbal extracts to reduce ocular oxidative stress. Thirty-two subjects participated in either an experimental group (wearing glasses containing herbal extracts) or a control group (wearing glasses without herbal extracts). During the ANT experiment, we collected electroencephalography (EEG) and electrooculography (EOG) data and measured button responses. In addition, electrocardiogram (ECG) data were collected before and after the experiments. The EOG results showed that the experimental group exhibited a reduced number of eye blinks per second during the experiment and faster button responses with a smaller variation than did the control group; this group also showed relatively more sustained tension in their ECG results. In the EEG analysis, the experimental group had significantly greater cognitive processing, with larger P300 and parietal 2–6 Hz activity, an orienting effect with neural processing of frontal area, high beta activity in the occipital area, and an alpha and beta recovery process after the button response. We concluded that reducing blue-light-induced oxidative stress with herbal extracts may be associated with reducing the number of eye blinks and enhancing attentive performance.

## 1. Introduction

Overexposure to the sun's ultraviolet (UV) rays has been associated with a number of eye problems, including pterygium, corneal degenerative changes, photokeratitis, and age-related cataracts [1–4]. Further, recent studies have revealed that blue light found in the visible spectrum and detectable by the human eye is correlated with oxidative stress [4–6] produced primarily by digital display technologies in typical working environments, including smartphones, tablets, laptops, monitors, and other devices. Thus, it is possible that reducing oxidative stress in human corneal epithelial cells will improve people's work performance. However, the association between oxidative stress and attention related to work performance has not yet been investigated.

Previous research [5] has shown that herbal extracts have potent protective effects against blue-light-induced oxidative stress, due to the fact that they increase the antioxidant enzymes heme oxygenase-1 (HO-1), peroxiredoxin-1 (Prx-1), catalase (CAT), and superoxide dismutase-2 (SOD-2) in corneal epithelial cells. Recently, Choi et al. [6, 7] also showed that herbal extracts can reduce the symptoms of dry-eye disease (DED).

In this study, we hypothesized that a reduction in oxidative stress may make people's eyes feel more comfortable and enhance their attentive performance. To measure specific work performance, we adopted the attention network test (ANT) experiment, as attention is related closely to work performance [8–10]. Functional magnetic resonance imaging (fMRI) studies [11, 12] and EEG studies [13, 14] using an ANT experiment have shown the anatomy of three different attentional networks. First, the alert state of attention changes in preparation to perceive a stimulus, which is critical for optimal performance in tasks that involve higher cognitive functions. Second, selective attention is used to choose among conflicting actions. Third, orienting attention is necessary for reference sensory objects. In this paper, we investigated how a reduction in oxidative stress using herbal extracts affected people's attentive performance by measuring brain oscillatory signals, behavioral responses, eye blinks, and heart rates.

## 2. Methods

### 2.1. Ethics Statement

The study protocol, informed consent, description of the study, and the data acquisition and processing methods were all approved by the Institutional Review Board of the Gwangju Institute of Science and Technology (number 20140602-HR-12-01-02) on June 2, 2014. This study was conducted at the Institute from July 28, 2014 to August 20, 2014.

### 2.2. Study Design

Thirty-two subjects (all healthy adults with a mean age = 23 ± 2.37 years, 14 females) signed a written informed consent to participate in the study. They were paid for their participation and were assigned randomly to either a control (placebo) or an experimental group, with 16 subjects per group. We prepared glasses containing either a herbal extract pad (experiment) or a nonherbal extract pad (placebo). The herbal extract pad in the glasses contained* Schizonepeta tenuifolia* var.* japonica Kitagawa*,* Angelica dahurica* Bentham et Hooker,* Rehmannia glutinosa* Liboschitz var.* purpurea Makino*, and* Cassia tora* L., which have antioxidant and anti-inflammatory effects that protect human corneal epithelial cells from oxidative stress [5–7]. The pad was made of polypropylene and viscos rayon. A pair of glasses with a pad-holding powder (0.4084 g) composed of mixed herbal extracts was used in this study. The extracts escaped, evaporated from the pad, and finally reached the eyes. During the ANT experiment, each subject in the experimental group wore a pair of glasses with a herbal extract pad, while the control group wore glasses with a pad that contained no herbal extracts in order to eliminate any biased results of the placebo effect. Each subject performed 14 experimental runs in the ANT experiment to collect 32-channel electroencephalogram (EEG) data. These experimental runs consisted of two one-minute baseline runs (one with eyes open, one with eyes closed), an electrocardiogram (ECG) run (three minutes), ten ANT runs, and an ECG run (three minutes).

### 2.3. Experimental Paradigm

**Figure 1:**
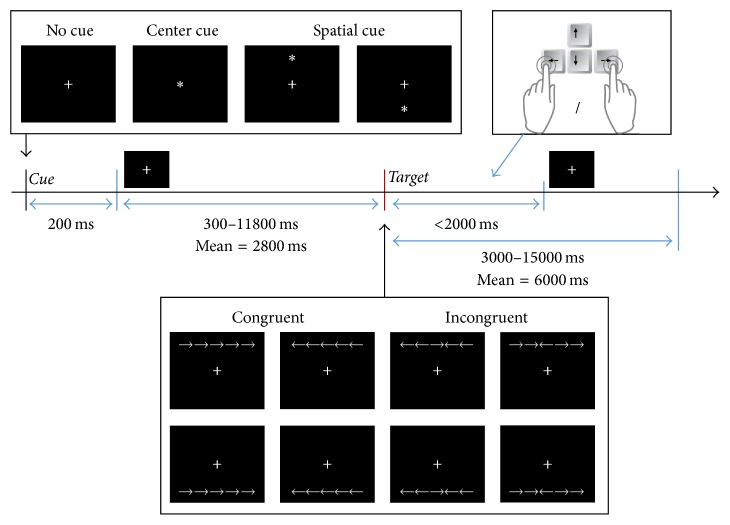
*One trial in the attention network (ANT) test*. A fixation cross appeared in the center of the screen at all times. Then, one of three cue types (none, center, and spatial) appeared for 200 msec. After a random interval (300–11800 msec, mean = 2800 msec), the target stimulus (upper/lower locations and left/right congruent or incongruent arrows) appeared until the subject responded. The duration of the presentation was 2000 msec at most. As soon as a subject pressed the button, the target stimulus disappeared. The next trial began after an intertrial interval that varied randomly and included a reaction time of 3000–15000 msec, mean = 6000 msec.

We employed the experimental paradigm introduced in Fan et al. [12] using an LCD monitor. As illustrated in Figure 1, one trial of the ANT experiment consisted of the presentation of three cues (none, center, and spatial) and two targets (congruent and incongruent) for a total of six conditions. For each trial, the subjects were instructed to press the left or right arrow key on the keyboard within two seconds, depending upon the direction of the center arrow among the five arrows. The color of the arrow was white (including blue light in the visible spectrum) and the background color was black. The center arrow in an incongruent target pointed in the opposite direction of the other four arrows (flankers). In comparison to the congruent target, the incongruent target might have required the subjects to engage in decision-making, thereby delaying button responses.

The center cue informed the subjects that a target would appear within 300–11,800 msec (Fan et al. [12]); however, the center cue did not indicate the position of the target, which could appear either in the upper or in the lower position. The spatial cue indicated the position where the target would soon appear. Therefore, a comparison of button responses between no cue and the center cue measured the alert effect, while the orienting effect was measured by the combination of the center and spatial cues. The conflict effect was measured by comparing the responses to the congruent and incongruent targets. In this study, 80 trials of EEG and key responses were collected for each of the four cues (none, center, upper spatial, and lower spatial), for a total of 320 trials per subject. There were 160 trials with the congruent/incongruent targets; after each cue, 40 congruent and 40 incongruent targets followed. There were 10 runs of 32 trials for each target. In each run, the 32 trial types (32 = 2 (no or center cue) × 8 (target conditions) + 2 × 2 (upper or lower spatial cues) × 4 (target conditions)) were presented in random order.

### 2.4. Data Recording

EEG data were acquired at 512 Hz using 32 active electrodes (Biosemi ActiveTwo system) attached to each subject's scalp according to the 10–20 international system. Reference channels were attached to both earlobes. ECG data were recorded for three minutes using a finger clip oximeter sensor (uBioClip v70 system, Biosense) before and after the experiment. Electrooculography (EOG) was used to record the number of eye blinks.

### 2.5. Preprocessing

EEG data were 1–45 Hz band-passed filtered and segmented into temporal windows ranging from 500 msec before target onset to 1000 msec after target onset for each trial. Mid- or high-levels of gamma activity (>45 Hz) were overlapped with muscle activity (20–30 Hz) [15] and were therefore filtered out. Components of eye blinking and movements were removed by independent component analysis (ICA) using the EOG data [16]. Bad trials with amplitudes exceeding ±100 *μ*V were also rejected [17].

### 2.6. Outcome Assessment

The primary measurements were event-related potential (ERP) and time-frequency (TF) map analyses of alerting, orienting, and conflict effects for each trial after target onset (Figure 1). First, we compared the ERP of the control and experimental groups (within-group analysis)—for example, no cue versus center cue (alerting), center cue versus spatial cue (orienting), and congruent target versus incongruent target (conflict), either in the experimental group or in the control group. Further, we calculated the difference in the ERP from two conditions; for example, no cue-center cue (alerting), center cue-spatial cue (orienting), and congruent target-incongruent target (conflict). Then we compared the differences of each pair between the experimental and control groups (between-group analysis). For TF map analysis, we calculated the power spectra of each channel under the following conditions: multitaper TF transformation based on multiplication in the frequency domain, Hanning taper, 1–30 Hz frequency of interest (30 bins), 0–1000 msec time of interest (21 points), and 7 cycles for each frequency bin. We conducted within- and between-group analyses with the same procedure as those for the ERP study.

After we found significant clusters, we created a cluster vector; each experimental subject's ERP and TF maps were projected to the cluster vector to quantify the attentional effect as a scalar value. Further, we projected the ERPs and TF maps to spatial or time axes to observe the attentional effect's location of the cluster.

The secondary measures were button responses, ECG, and EOG. First, as behavioral data analysis for each subject, we analyzed means and standard deviations of response time (RT) for all 320 trials. For ECG data, we compared beats per minute (BPM) before and after the ANT experiment. Lastly, we counted the number of eye blinks using EOG during 10 runs of the ANT experiment.

### 2.7. Statistical Analyses

To avoid the multiple comparisons problem (MCP) in EEG analysis, we performed a nonparametric cluster-based permutation test [18, 19] using the Fieldtrip toolbox [20]. Adjacent spatiotemporal (ERP analysis) or spatiospectrotemporal (TF map analysis) points can be clustered according to their significance and the *p* value of clusters (cluster-level statistic) can be estimated using a Monte-Carlo simulation. Assuming an individual point in a cluster with a type I error of *p* < 0.05, a cluster-based permutation test revealed that significance could only be claimed if more than two contiguous original points in a cluster reached significance, regardless of cluster shape. Thus, the evaluation of the differences between two conditions for each of the samples in clusters can be replaced by a single comparison using cluster-level statistics, which solves the MCP. We used the *t*-statistic for dependent samples for within-group analyses and the *t*-statistic for independent samples for between-group analyses, all at *p* < 0.05. The minimum size of a cluster was two. A neighboring channel was defined as demonstrating spatial adjacency within 4 cm. We conducted a Monte-Carlo simulation with 1000 iterations using a cluster-based permutation test to approximate the *p* value of each cluster in the spatiotemporal or spatiospectrotemporal space. The cluster-level statistics were calculated using the sum of the *t*-values in the cluster. The significance of each cluster was calculated in spatiotemporal (matrix of size (# of channels) × (# of time samples)) or spatiospectrotemporal space (matrix of size (# of channels) × (# of frequency bins) × (# of time samples)), with a time range of 0–1000 msec after the onset of the target stimulus (Figure 1). The procedure for the cluster-based permutation test for ERP (depicted in Figure 3) is as follows.

(0) We calculated the average ERP over 50 trials. Each subject had four ERPs corresponding to the three images. For example, if we compared the ERPs over subjects between circle versus bat, the *t*-value was calculated for each spatiotemporal point because the ERP data are considered a channel by time matrix.
(1) For the cluster-based permutation test, we used all *t*-values with a *p* value < 0.05. Here, the *p* value was calculated by a parametric *t*-test and was not corrected statistically. In addition, we summed all the positive or negative *t*-values within the clusters separately. The summed values constituted the cluster-level statistics, for which we approximated the significance.
(2) The selected *t*-values were clustered based on spatiotemporal adjacency. The minimum size of a cluster was set to two points. A neighboring channel was defined as having spatial adjacency within 4 cm [19]. We note that channel switching makes no difference in our analysis since neighboring channels are determined according to their spatial adjacency.
(3) We shuffled the conditioned trials, divided the shuffled trials into two datasets, and then conducted a *t*-test for the two sets to obtain a *t*-value map.
(4) We used a Monte-Carlo simulation of 1000 iterations of Step (3) to approximate the cluster-level *p* value.
(5) We took the largest of the cluster-level statistics for each permutation result and obtained 1000 values of the cluster-level statistics.
(6) We constructed a histogram of the 1000 values of the cluster-level statistics, and a probability density function (PDF) was calculated to estimate the cluster-level *p* values. The input for the PDF was the cluster-level statistic from the first step, while the output was a *p* value for each cluster-level statistic. Thus, the cluster-level *p* values were corrected and approximated by a cluster-based permutation test, because multiple comparisons were transformed into a single cluster-level comparison.

After the cluster-based permutation test, we used each significant cluster as a feature extraction filter in ERP analysis, as follows:

(1) $$\begin{array}{c} q_{ERP}=\underset{s\in S}{\sum }\;\underset{t\in T}{\sum }w_{st}X_{st}. \end{array}$$

Here *s* and *t* indicate spatial and temporal indices, respectively; *S* and *T* are sets of whole channels and time points in this analysis, respectively. *X*_st_ is the average of the spatiotemporal data over trials in one subject. *w*_st_ is a spatiotemporal weight representing a value of 0 or 1. For the spatiotemporal point (*s*, *t*) in the cluster, *w*_st_ is 1 and 0 otherwise. Furthermore, in order to observe spatial and temporal features separately, the values in a cluster were summed over each channel and time point as follows:

(2) $$\begin{array}{c} v_{ERP\_S}=\underset{t\in T}{\sum }w_{st}X_{st}. \end{array}$$

Here *v*_ERP_S_ is a feature vector. The size of *v*_ERP_S_ is the number of channels. By using (2) we observe which channels are discriminative.

For TF analysis, the frequency bin index *f* ∈ *F* is added as follows:

(3) $$\begin{array}{c} q_{TF}=\underset{s\in S}{\sum }\;\underset{f\in F}{\sum }\;\underset{t\in T}{\sum }w_{sft}X_{sft}. \end{array}$$

Here *X*_*sft*_ is the averaged TF map over trials for one subject. Similar to ERP analysis, we summed the values in a cluster to extract spatial, spectral, and temporal features as follows:

(4) $$\begin{array}{c} v_{TF\_S}=\underset{f\in F}{\sum }\;\underset{t\in T}{\sum }w_{sft}X_{sft}. \end{array}$$

Here *v*_TF_S_ is a feature vector. The size of *v*_TF_S_ is the number of channels. By using (4) we could observe which channels are discriminative.

Furthermore, extracted spatial features of two conditions were statistically tested for each channel over subjects to observe discriminative channels or brain areas. Estimated *p* values were corrected by false discovery rate (FDR). Individual topographies were presented in the supplemental material, as shown in Figures S1–S15 in Supplementary Material available online at http://dx.doi.org/10.1155/2016/4292145.

## 3. Results

### 3.1. Behavioral Data

**Figure 2:**
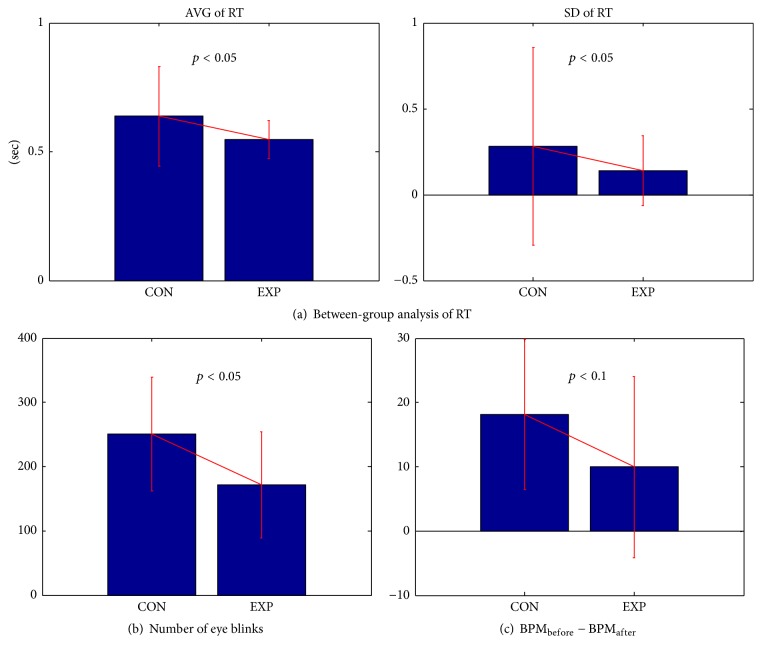
*Results of between-group analysis of button response time (RT), EOG, and ECG.* (a) Comparisons of the average and standard deviations of the RTs between the two groups, (b) averages of the number of eye blinks for each group in each run, and (c) boxplots of differences in BPM between the groups before and after the ANT experiment.

**Figure 3:**
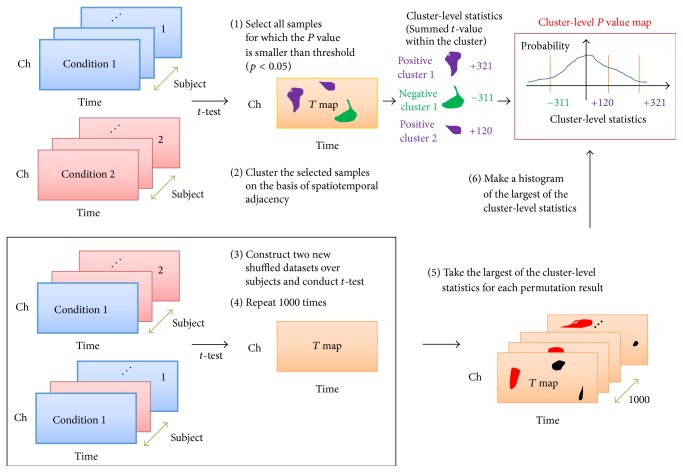
*Procedure for cluster-based permutation test in ERP (spatiotemporal data) analysis*. First, *t*-tests were conducted for each channel and time point for all subjects. Then, we obtained an uncorrected spatiotemporal *t*-value map. Second, we clustered the selected samples in connected sets based on spatiotemporal adjacency. Positive or negative *t*-values in a cluster were summed separately. Third, we permuted the ERP without condition; the condition was the circle, star, or bat image. After the permutation, we performed *t*-tests for each channel and time point. Fourth, we iterated the third procedure 1000 times to obtain 1000 *t*-value maps. Fifth, we determined the largest of the cluster-level statistics for each of the 1000 *t*-value maps. Sixth, we constructed a histogram of the largest values and a probability density function based on the cluster-level statistics. Finally, we obtained a *p* value that was approximated and corrected by this nonparametric permutation test from the probability density function. Reprinted from [18]. Copyright 2016 by the Neurosignals.

RTs were collected for each trial. The means and standard deviations (SDs) of the RTs for 320 trials were estimated to compare the control and experimental groups. The means and SDs of the RTs in the experimental group were significantly smaller than were those in the control group (*p* < 0.05; Figure 2(a)). Thus, the experimental group exhibited faster and more robust RTs over trials than did the control group.

### 3.2. EOG and ECG Data

The average numbers of eye blinks per run were 250.93 ± 11.81 and 171.56 ± 7.47 for the control and experimental groups, respectively. The experimental group exhibited significantly fewer eye blinks than did the control group, as shown in Figure 2(b). Specifically, after an insignificant first run, the control and experimental groups began to show significant differences (*p* < 0.05) during the second run. For ECG, the BPM between measurements taken before and after the ANT differed (*p* < 0.10), as shown in Figure 2(c).

### 3.3. Results of Event-Related Potential (ERP) Analysis

**Figure 4:**
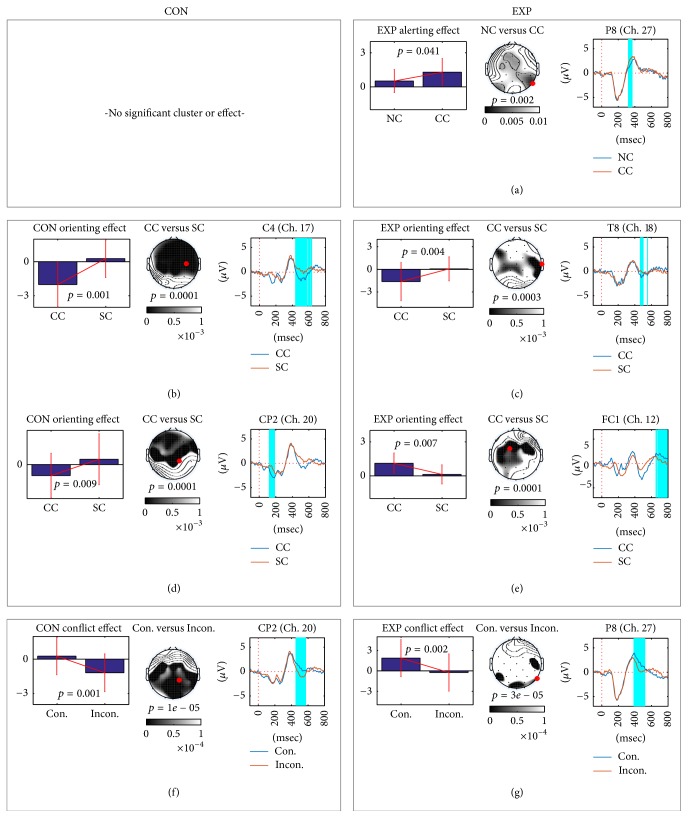
*Within-group comparisons of two target ERPs on the control and experimental group*. We used the cluster as a feature extraction filter introduced in the method section. (a)–(g) We plotted results of significant clusters (*p* < 0.05). For each cluster, we showed a grand averaged bar graph, a *p* value topography, and two target ERPs over subject from control or experimental group. Bar graphs show quantified attention effect (alerting, orienting, and conflict). Topography shows significant *p* values (FDR corrected) and discriminative area between extracted spatial features of two conditions. Small black dots mean channel location and red dots are the most discriminative channel location (the smallest *p* value). Lastly, cyan-colored areas show the range of the significant cluster in the ERPs from the red dotted channel in the topography. We could not find any significant cluster or effect from the alerting condition in the control group.

In the within-group ERP analysis, we found a significant cluster in the experimental group only for the alerting effect. The quantified values (1) of the experimental group demonstrated larger P300 responses for the center cue than those for no cue, as shown in Figure 4(a). P8 channel showed the biggest alerting effect in the experimental group. As for averaged topographies (2) over experimental subjects, P300s for the frontal and right parietal areas were higher than were those for no cue. The most discriminative channel was P8 channel between no cue and center cue (red mark in Figure 4(a)). The averaged P300 amplitudes of center cue responses were larger than those of the responses to no cue.

In orienting effect analysis, both control and experimental groups showed bigger N400 responses in center cue than spatial cue, as shown in Figures 4(b) and 4(c). The topographies showed that the difference of N400 was located around central and temporal area; discriminative channels were detected on central area (C4 or T8 channel). On the other hand, the control group showed orienting effect on central area including CP2 channel (the most discriminative channel) within 100–200 msec, while the experimental group showed the difference between center cue and spatial cue in the frontal and central area. The cluster of Figure 4(e) in CP2 channel ranged around 600–800 msec. The spatial cue showed smaller potential than the center cue.

Both the control and experimental groups showed similar conflict effects in ERP analysis, as shown in Figures 4(f) and 4(g). N400 responses of an incongruent target were smaller than those of the congruent target. The temporal patterns of the control and experimental groups were similar to each other. This pattern is also known as inhibitory process for incongruent target [14].

**Figure 5:**
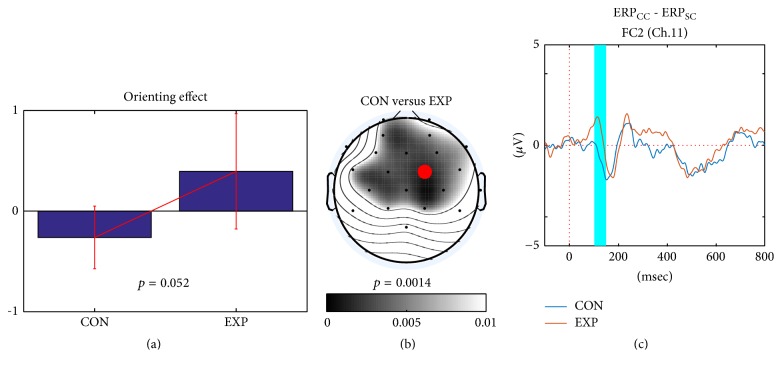
*Between-group comparisons of two differential ERPs (control and experimental groups) on orienting effects*. (a) The orienting effect in the experimental group was greater in the frontal and central areas (b) at approximately 100–150 msec (cyan-colored area) than it was in the control group (c). We could not find any significant clusters or effects from alerting and conflict conditions in the between-group comparisons. ERP_CC_ and ERP_SC_ mean the target ERP of center cue and spatial cue.

In the between-group ERP analysis, both control and experimental groups showed differences only in the orienting effect between target ERPs after the center and spatial cues, as shown in Figure 5. The estimated *p* value was not significant (*p* = 0.052). The quantified orienting effect in the experimental group was greater than that in the control group (Figure 5(a)) and was measured in the frontal area (Figure 5(b)) around approximately 100–150 msec (Figure 5(c)). The location of the orienting effects varied slightly over the frontal and central areas. The most discriminative channel was FC2 channel (red mark in Figure 5(b)).

### 3.4. Results of Time-Frequency Map Analysis

**Figure 6:**
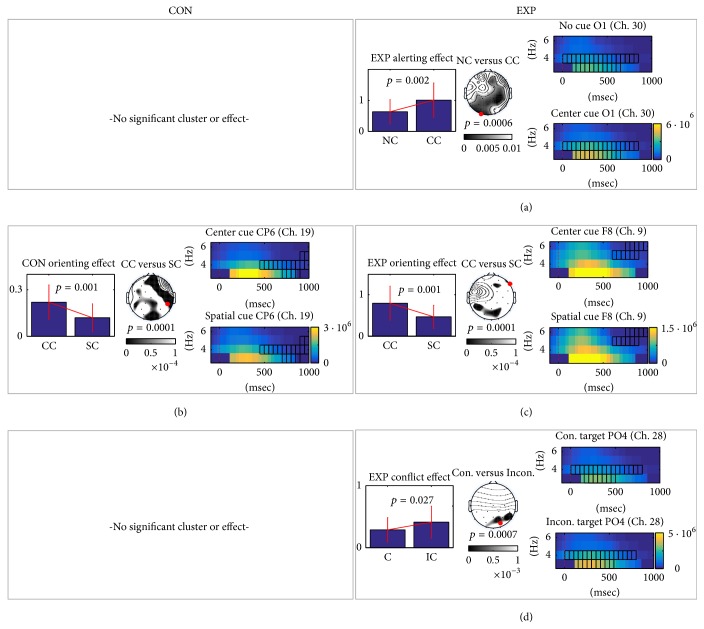
*Within-group comparisons of two target TF maps in control and experimental group.* (a)–(d) We plotted results of significant clusters (*p* < 0.05). Interestingly, all the clusters in the within-group analysis were detected around high delta and low theta band (2–6 Hz). For each cluster, we showed a grand averaged bar graph, a topography, and two target TF maps over subject from control or experimental group. Bar graphs show quantified attention effect (alerting, orienting, and conflict). Topography shows significant *p* values (FDR corrected) and discriminative area between extracted spatial features of two conditions. Small black dots mean channel location and red dots indicate the most discriminative channel location. Lastly, we showed TF maps from the red dotted channel. Black squares in the TF map mean the cluster location in that channel. All the 2–6 Hz activities were peaked around 400 msec and then decreased. Commonly, significant *p* values were detected around parietal area for all comparisons. We could not find any significant cluster or effect from alerting and conflict conditions in the control group.

Interestingly, the within-group TF analysis showed that all significant clusters were detected in high delta and low theta bands (2–6 Hz) around parietal area. The power spectrums peaked at 3 Hz. All the 2–6 Hz activities were peaked around 400 msec in terms of grand averaged behavior and then decreased, as shown in Figure 6. At first, the quantified effect (3) of the experimental group demonstrated significantly greater 2–6 Hz activity (*p* < 0.05) for the alerting (the center cue versus no cue), as shown in Figure 6(a). The averaged topography of alerting effect showed that the right frontal, central, parietal, and left occipital areas were active after the center cue; however, the most significant channel was located on occipital area (red mark in Figure 6(a)). The experimental group showed the alerting effect only, while an alerting effect for the control group was not found. In orienting effect, both control and experimental groups showed reduced 2–6 Hz activity for spatial cue compared with center cue around 500 to 1000 msec (Figures 6(b) and 6(c)). In conflict effect, the incongruent target showed greater 2–6 Hz activity in the experimental group, while the control group did not show any significant cluster (Figure 6(d)).

**Figure 7:**
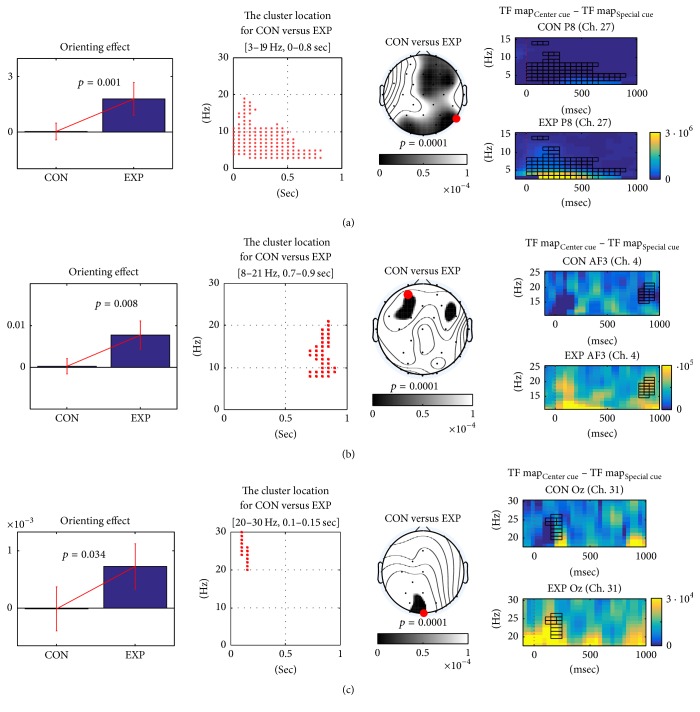
*Between-group comparisons on orienting effects of two differential TF maps (control and experimental groups)*. We found three significant clusters only on orienting effects. We plotted results of significant clusters (*p* < 0.05). For each cluster, we showed a bar graph, cluster location in spectrotemporal space, a topography, and two target TF maps over subject from control or experimental group. We marked the channel location in red next to the TF maps. Black squares mean cluster locations in TF map of red dotted channel. (a) The experimental group showed greater 3–19 Hz activity at approximately 400 msec, (b) the experimental group showed greater alpha and low beta (8–20 Hz) activity at approximately 800 msec, and (c) the experimental group showed greater high beta activity (20–30 Hz) within 100–150 msec.

Further, between-group TF analysis showed significant results only in orienting effect. We could not find any significant clusters in alerting and conflict effects, as shown in Figure 7. The experimental group showed three significant clusters (*p* < 0.05) in the orienting effect (Figure 7), while the control group showed no orienting effect in the differential TF analysis (target TF maps of center cue versus target TF maps of spatial cue). Similar to the within-group TF analysis, we observed significant 2–6 Hz activity (*p* < 0.01) at approximately 400 msec (Figure 7(a)). The most discriminative channel between center and spatial cue was P8 channel. Significant alpha (8–14 Hz) and low beta (8–21 Hz) activities were also observed (*p* < 0.05) at approximately 800 msec over the frontal areas (Figure 7(b)). The most discriminative channel was AF3 channel. Lastly, we observed significant high beta activity (20–30 Hz) (*p* < 0.05) within 50–200 msec over the occipital areas including Oz channel (Figure 7(c)), while the control group showed small orienting effects (difference between target TF maps of center and spatial cues).

## 4. Discussion

### 4.1. Reduced Eye Blinks

Eye blinking was significantly lower in the experimental group than in the control group, as shown in Figure 2(b). According to the literature [21, 22], eye blinking is useful in measuring eye fatigue, because it may supply tears and prevent drying. Eye blinking is therefore positively correlated with eye fatigue, as it is the body's attempt to relieve the negative effects of fatigue. Previous human and animal studies have shown that herbal (or medicinal plant) extracts from* Schizonepeta tenuifolia* var.* japonica Kitagawa*,* Angelica dahurica* Bentham et Hooker,* Rehmannia glutinosa* Liboschitz var.* purpurea Makino*, and* Cassia tora* L. increased the antioxidant enzymes heme oxygenase-1 (HO-1), peroxiredoxin-1 (Prx-1), catalase (CAT), and superoxide dismutase-2 (SOD-2) in corneal epithelial cells that protect against oxidative stress induced by blue light [5–7]. Further, previous human and animal studies [6, 7] have shown that herbal extracts are an effective treatment for symptoms of dry-eye disease. Thus, the herbal extracts reduced eye blinks during the ANT experiment by reducing oxidative stress on corneal epithelial cells.

### 4.2. Rapid and Robust Button Response Time

We observed that the button responses in the experimental group were statistically significantly faster and more robust (smaller SD) than were those in the control group. This evidence supports the idea that the reduction of oxidative stress induced by herbal extracts may enhance attentional performance. We found additional evidence of increased attentional processing in the ECG, EOG, and ERP analyses, as follows.

- Eye blinking in the experimental group was significantly lower than in the control group, suggesting reduced eye fatigue. Thus, we expected that the experimental group would maintain better eye condition during the ANT experiment than would the control group.
- For the ECG feature, BPM decreased in the control group, as shown in Figure 2(c); however, there was a relatively small difference in the ECGs before and after the ANT in the experimental group. Higher BPM indicates a state of tension [23, 24]. Thus, we inferred that the control group relaxed after the ANT experiment, while the experimental group maintained its attention during the ANT experiment.
- Although the experimental group showed a significant alerting effect in ERP analysis, the control group did not, as illustrated in Figure 4(a). The alerting effect in the experimental group appeared clearly only in the frontal and parietal areas at approximately 320–390 msec. The frontal and parietal areas are related to maintaining an alert state, as reported in [11, 12]. Considering the ERP and eye-blinking behaviors, the control group may have experienced eye discomfort as time passed, rendering subjects unable to attend to the task well. However, the experimental group remained alert and showed greater cognition (generally, the P300 component results from cognition) in the center cue due to eye comfort.

### 4.3. Parietal 2–6 Hz Activity

We found greater parietal 2–6 Hz activity in the experimental group than in the control group from the alerting and conflict effects in the within-group analysis and the orienting effect in the between-group analysis. The time windows observed (approximately 400 msec) were similar in both groups, as shown in Figures 6 and 7. The 2–6 Hz activity observed in the parietal area was also similar in terms of grand averaged patterns. The 2–6 Hz frequency band covers high delta and low theta bands. Delta activity is involved in deep-sleep stage 3 in nonrapid eye movement sleep [25] and slow-wave sleep [26]. Theta activity is known to indicate a meditative [27] or sleep state [28], memory formation, and working memory [29, 30]. However, it was difficult to find studies on parietal delta or theta activities. Because of the high BPM and reduced eye blinks in the experimental group, we believe that the parietal 2–6 Hz activity was not related to a meditative or sleep state. In addition, the ANT experiment was not related to memory, while frontal theta activities have been reported in memory studies. Because of the time window observed (approximately 400 msec) in the cortical area (parietal area), it is believed that the parietal 2–6 Hz activity was related to the P300 component, which was also reported in [31]. They found theta activity during the P3a time interval. Here, we also found that most of the P300 components occurred within 300–500 msec, which is the same as the interval of parietal 2–6 Hz activity; the frequency of the P300 component was within the theta band. Lastly, P300 is also known to be located in the parietal area [31, 32]. Therefore, it is inferred that parietal 2–6 Hz activity may be associated with the frequency of P300.

### 4.4. Greater Orienting Effect in Experimental Group

In the between-group ERP analysis of Figure 5, the orienting effect at approximately 100–150 msec was significant primarily in the superior frontal cortex (Fz channel), which is related to preparing for goal-directed selection [33]. The positive differential value of ERPs demonstrated that the target ERP following a response to the center cue was greater than that after the spatial cue. After the spatial cue, subjects did not have to shift their attention to an upper or lower position, because the spatial cue already gave the position of the target stimulus. However, with the center cue, the subjects had to shift their focus after the presentation of the target stimulus when the target location appeared suddenly. It is believed that the orienting effect at approximately 100 msec in the superior frontal cortex is related to neural processing in the frontal eye fields (FEF) [33, 34]. Because the experimental group showed a larger orienting effect than did the control group, this indicates that the target ERP of the center cue responses in the experimental group occurred robustly over all trials because ERP was averaged.

In the between-group TF map analysis of Figure 7(c), we observed high beta activity (20–30 Hz) in the left central and occipital area within 50–200 msec after target onset. Fan et al. [13] reported that the high frequency activities (>20 Hz) in the orienting effect occur in the left/right fusiform gyrus, right superior parietal lobule, and left/right postcentral gyrus within 400 msec after target onset. This indicates that the experimental group showed a known orienting effect more clearly than did the control group. One possible reason for this result is that the experimental subjects exhibited fewer eye blinks than did those in the control group, so that it was easier for the former to concentrate on the ANT task. Importantly, the interval between the cue and target stimulus was randomized (300–11800 msec, mean = 2800 msec). Thus, reduced eye blinks can lead to more rapid and robust responses to the stimulus, as shown in the button responses.

Lastly, we found alpha and low beta activities (8–20 Hz) at approximately 800 msec, as shown in Figure 7(b). Alpha and low beta activities at 800 msec are related to postprocessing after the button responses, most of which were made before 800 msec. Event-related synchronization (ERS) is known to be a recovery process after cortical processing or event-related desynchronization (ERD) [35]. Although we did not observe a significant ERD before the button response, alpha and low beta activities are related to ERS and the recovery process of ERD.

### 4.5. Group Analysis and Intersubject Variability

All comparisons conducted in this work were group analysis. This group analysis represents overall statistical difference between conditions, but it could not represent intersubject variability in a detailed manner. For example, within-group analysis of Figure 4(a) represented the idea that P300 components of center cue (CC) condition were statistically larger than those of no cue (NC) condition, but it could not mean that for all subjects P300 components of CC were larger than those of NC condition. As illustrated in Figure S1, we observed that CC components were not larger than NC components for some subjects. Similar to Figure S1, we plotted all individual topographies (Figures S1 to S15) over 15 comparisons. This intersubject variability may be critical in our group analysis. In order to reduce intersubject variability, the normalization for each subject may be applicable in that it may prevent the dominant effect of a few subjects having far big activities. However, we found that the normalization might lose critical discriminative information between conditions. Other better approaches to reduce intersubject variability in group analysis should be sought or developed in the future work. Anyway, this work has some limitation in this respect. To make our group analysis more comprehensive, we presented spatial distribution of *p* values; it represents which channel is the most discriminative between two conditions or two groups. Assuming two distributions are Gaussian distributions, bigger difference between two means of the distributions or smaller summation of two standard deviations of the distributions may yield smaller *p* value. Thus, it is believed the channel selected by the *p* value may be a reasonable robust feature over subjects.

### 4.6. Effect of Reduced Oxidative Stress on Attentive Performance

**Figure 8:**
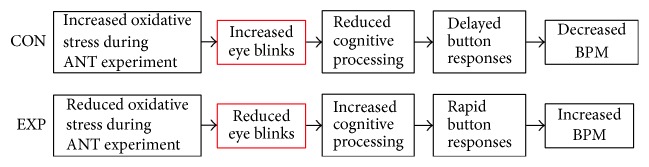
*Possible mechanism of effect of herbal extracts*. Reduced number of eye blinks could be the main effect of herbal extracts. The reduced eye blinks may facilitate the subject's concentration during the ANT experiment.

We would like to emphasize that herbal extracts did not affect the central nervous system (CNS) or attention network directly but affected only the subject's eyes, ultimately reducing eye blinks.  Figure 8 shows a possible mechanism of effect of herbal extracts. Due to the random interval (300–11800 msec, mean = 2800 msec) between cue and target onset, the experimental group that showed reduced eye blinks was able to respond more quickly and robustly. Therefore, we checked the number of eye blinks per second within the interval between the cue and target in 320 trials and found significant differences between the groups.

Initially, there was a significant between-group difference in the total number of blinks per second (*p* < 0.01), as shown in last column in Table 1. Further, the interval after the center and spatial cues showed a significant difference (*p* < 0.01). In the control group, the averages of eye blinks of center and spatial cues were bigger than after no cue. On the other hand, the eye blinks after center and spatial cues in the experimental group were smaller than after no cue. These results explain why we found the three significant clusters only in the orienting attention effect in the between-group analysis. The experimental group's ability to keep their eyes open helped them respond to the target faster than those in the control group.

## 5. Conclusions

We investigated whether herbal extracts enhanced subjects' attentive performance to visual cues and their subsequent button responses; in the experimental group, herbal extracts for DED were effective in reducing the number of eye blinks before target onset. As such, the experimental group showed more rapid and stable button responses than did the control group. In addition, the experimental group showed greater cognitive processing, including P300 and parietal 2–6 Hz activity, and a greater orienting effect, such as the neural processing of frontal area in the ERP results, high beta band activity in the left central and occipital area, and alpha and low beta recovery after the button press. Therefore, from the behavioral, ECG, EOG, and EEG results, our study indicates that attentive performance might be enhanced when blue-light-induced oxidative stress is reduced.

## Supplementary Material

There are 15 binary comparisons including within- and between- group analyses in this paper. To investigate inter-subject variability, individual topographies were presented, as shown in Figure S1-S15.

## Figures and Tables

**Table 1:** * Averaged number of eye blinks per second within the interval between cue and target onset (300–11800 msec, mean = 2800 msec) over 32 subjects*. The experimental group showed a significantly lower number of eye blinks than did the control group. Each cue condition included 80 trials, for a total of 320 trials.

	No cue	Center cue	Spatial cue	Total (blinks/sec)
CON	0.70 ± 0.24	0.72 ± 0.27	0.72 ± 0.26	0.71 ± 0.25
EXP	0.45 ± 0.30	0.43 ± 0.29	0.42 ± 0.30	0.44 ± 0.30
*p* value	0.02	0.005	0.008	**0**.**009**
