# Supplementary material for: Herbal Extracts That Reduce Ocular Oxidative Stress May Enhance Attentive Performance in Humans
Source: Comput Intell Neurosci. 2016 Dec 20;2016:4292145. doi: 10.1155/2016/4292145 (PMC5206474; doi:10.1155/2016/4292145)
Supplement: Supplementary file 1 — There are 15 binary comparisons including within- and between- group analyses in this paper. To investigate inter-subject variability, individual topographies were presented, as shown in Figure S1-S15. [file 4292145.f1.pdf]

Supplemental Material for the Manuscript:

Herbal extracts that reduce ocular oxidative stress may enhance attentive performance in humans

Hohyun Cho,[a] Moonyoung Kwon,[a] Hyojung Jang,[a] Jee-Bum Lee,[b] Kyung Chul Yoon,[c] and Sung Chan Jun[a]*

[a]School of Information and Communications, Gwangju Institute of Science and Technology, Gwangju, Korea

[b]Department of Dermatology, Chonnam National University Medical School and Hospital, Gwangju, Korea

[c]Department of Ophthalmology, Chonnam National University Medical School and Hospital, Gwangju, Korea

*Corresponding author email: scjun@gist.ac.kr (SCJ)

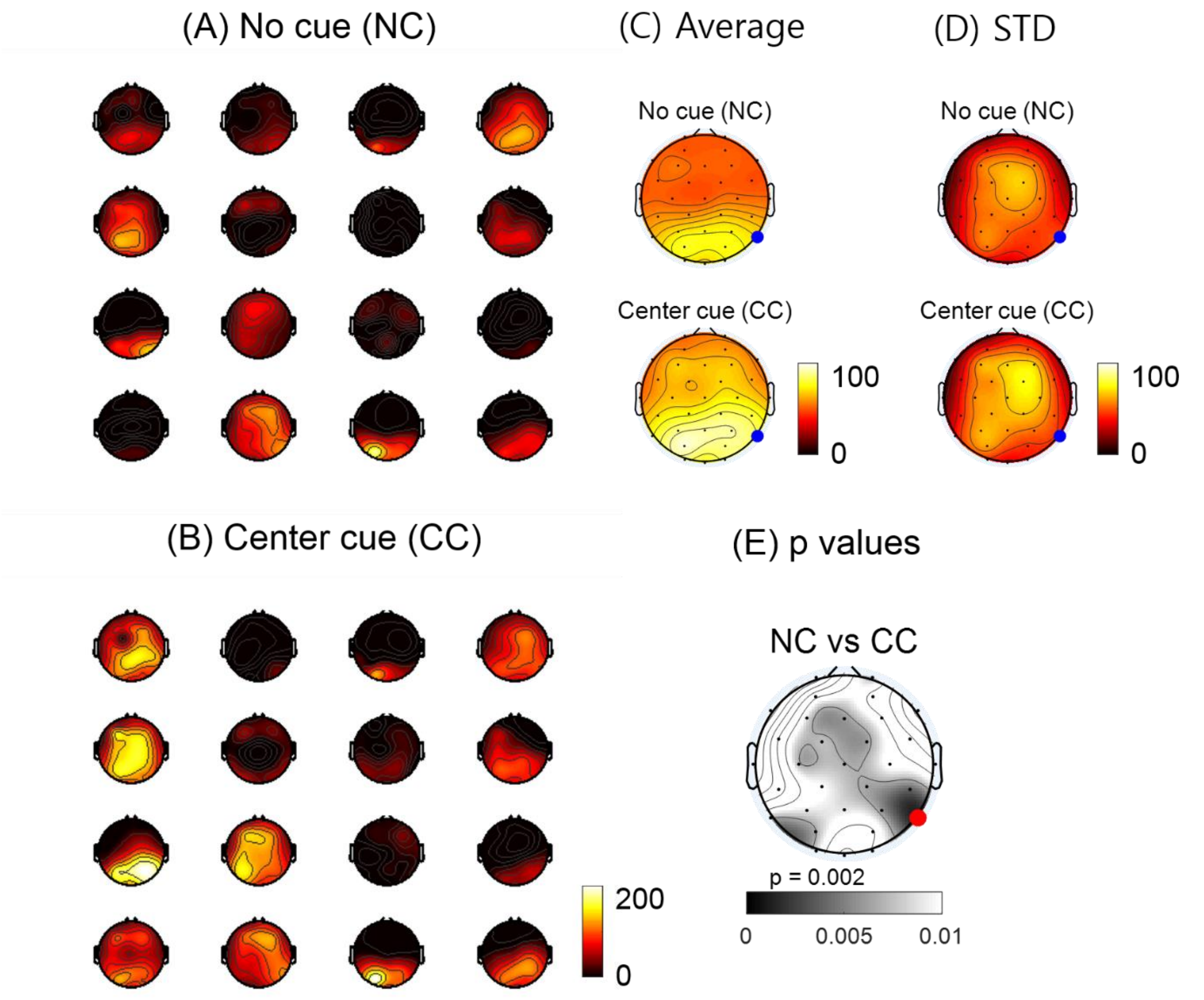

Figure S1 Normalized individual topographies for each subject (supplemental information for Figure 4A). Sixteen topographies of (A) no cue and (B) center cue are plotted. (C) Averaged topographies of no cue and center cue. (D) Topographies of standard deviation of no cue and center cue. (E) Topography of p values (corrected by FDR). Blue or red marked channel location (P8) had the smallest p value (FDR corrected p=0.002). Averaged topographies showed notable differences in parietal and occipital areas. Standard deviations showed the relative high variations in frontal, central, and parietal areas. However, p-value topography showed different pattern from averaged one. P8 channel showed the highest significance between conditions.

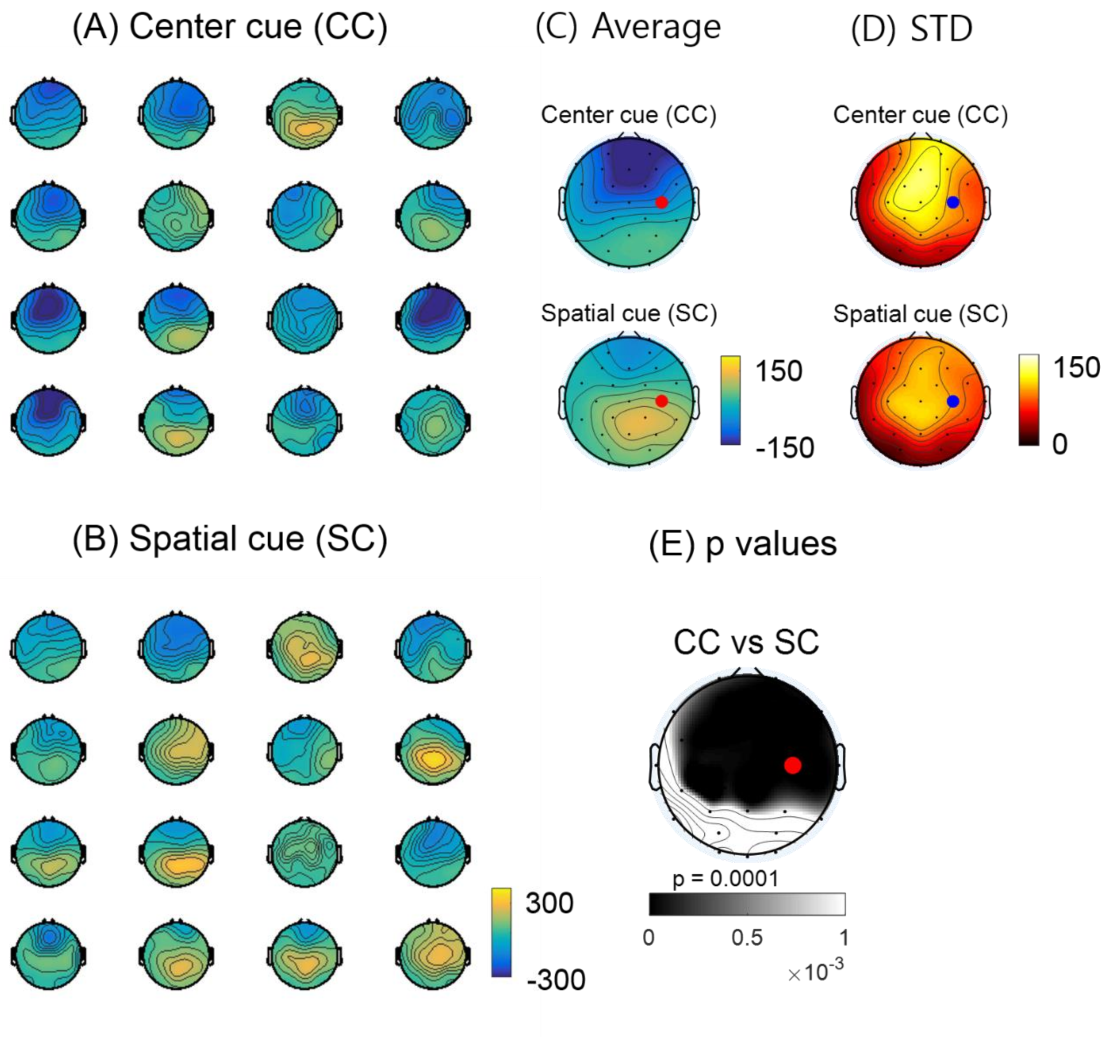

Figure S2 Normalized individual topographies for each subject (supplemental information for Figure 4B). Sixteen topographies of (A) center cue and (B) spatial cue are plotted. (C) Averaged topographies of center cue and spatial cue. (D) Topographies of standard deviation over sixteen subjects. (E) Topography of p values (corrected by FDR). Red or blue marked channel location (C4) had the smallest p value (FDR corrected p=0.0001).

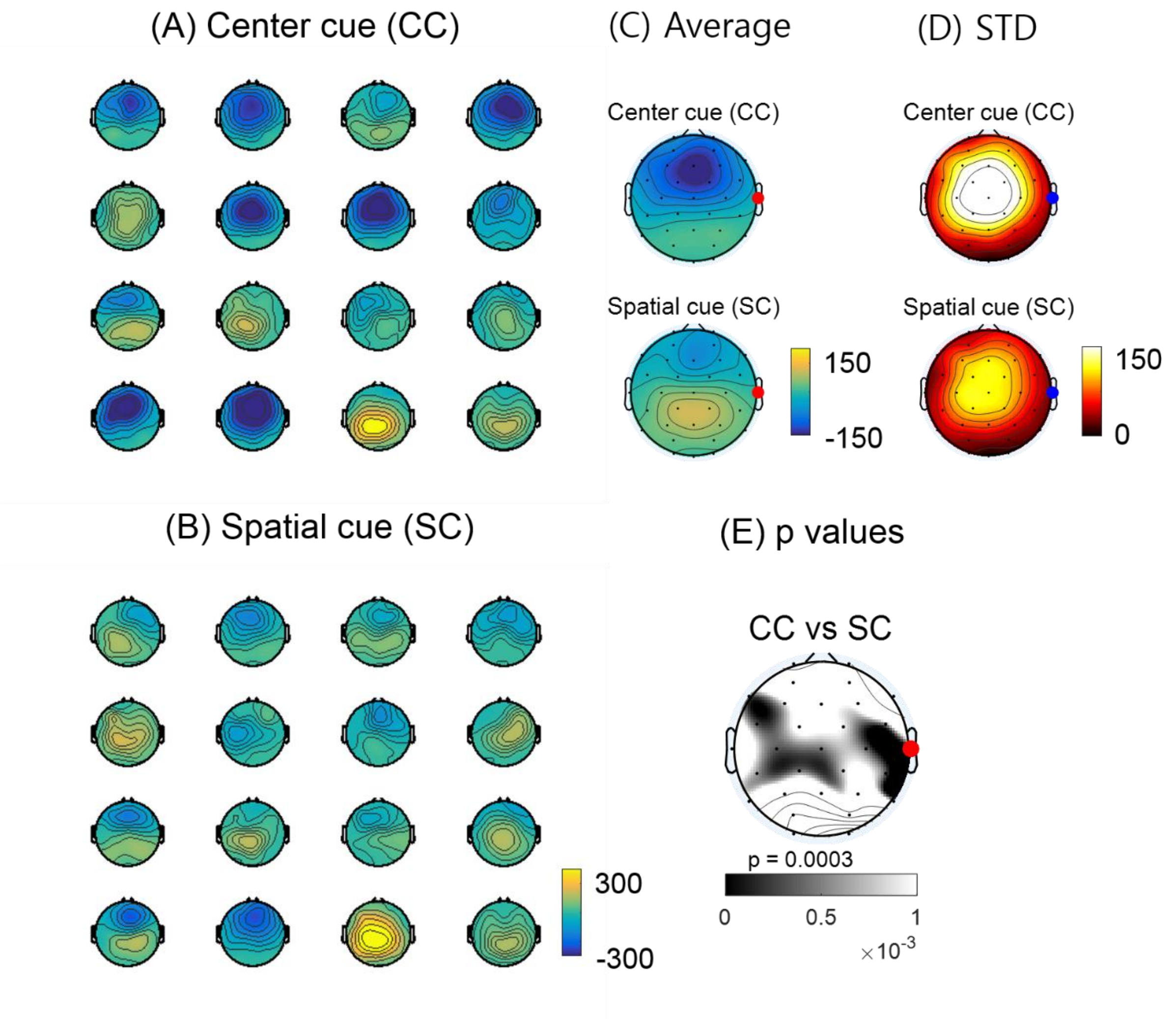

Figure S3 Normalized individual topographies for each subject (supplemental information for Figure 4C). Sixteen topographies of (A) center cue and (B) spatial cue are plotted. (C) Averaged topographies of center cue and spatial cue. (D) Topographies of standard deviation over sixteen subjects. (E) Topography of p values (corrected by FDR). Red or blue marked channel location (T8) had the smallest p value (FDR corrected p=0.0003).

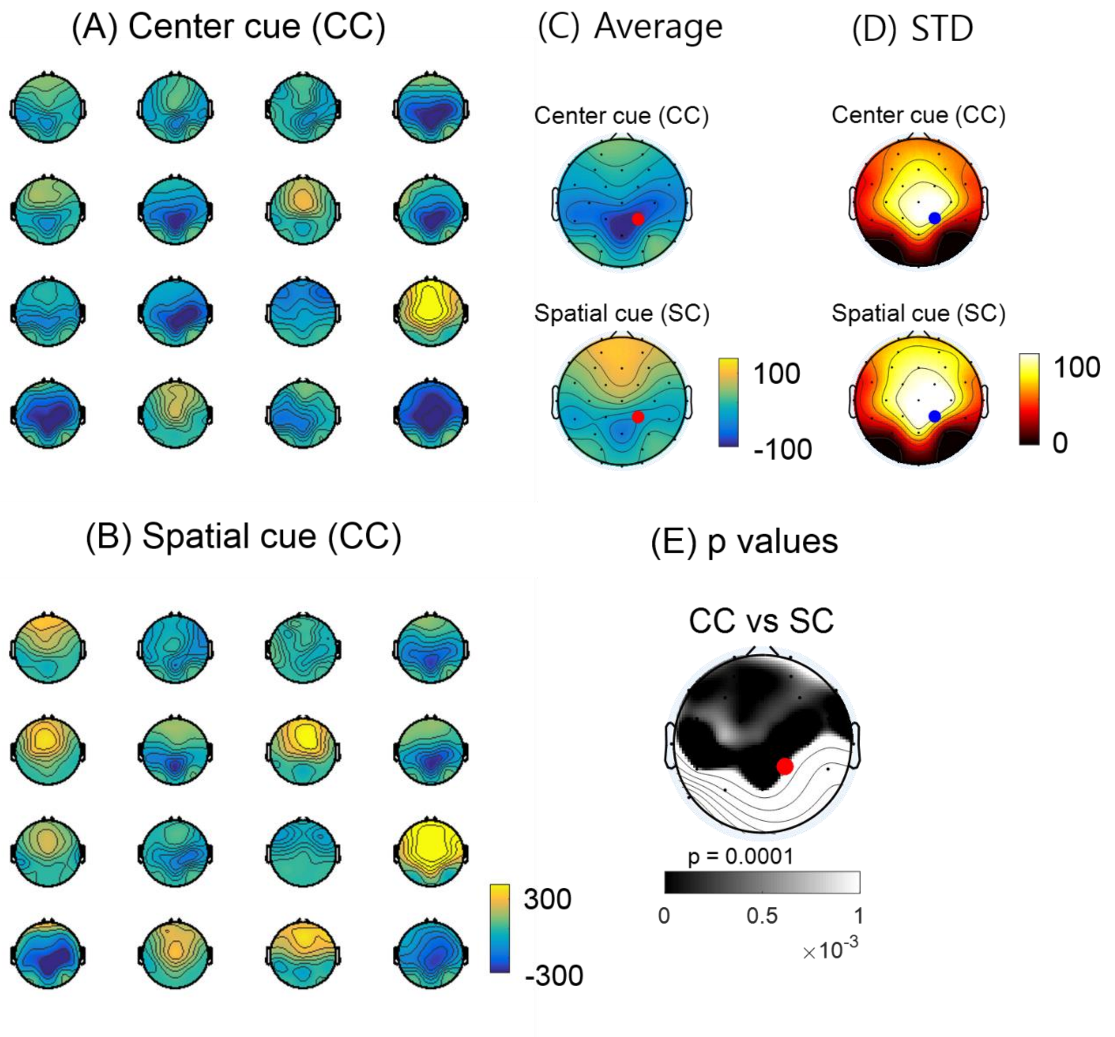

Figure S4 Normalized individual topographies for each subject (supplemental information for Figure 4D). Sixteen topographies of (A) center cue and (B) spatial cue are plotted. (C) Averaged topographies of center cue and spatial cue. (D) Topographies of standard deviation over sixteen subjects. (E) Topography of p values (corrected by FDR). Red or blue marked channel location (CP2) had the smallest p value (FDR corrected p=0.0001).

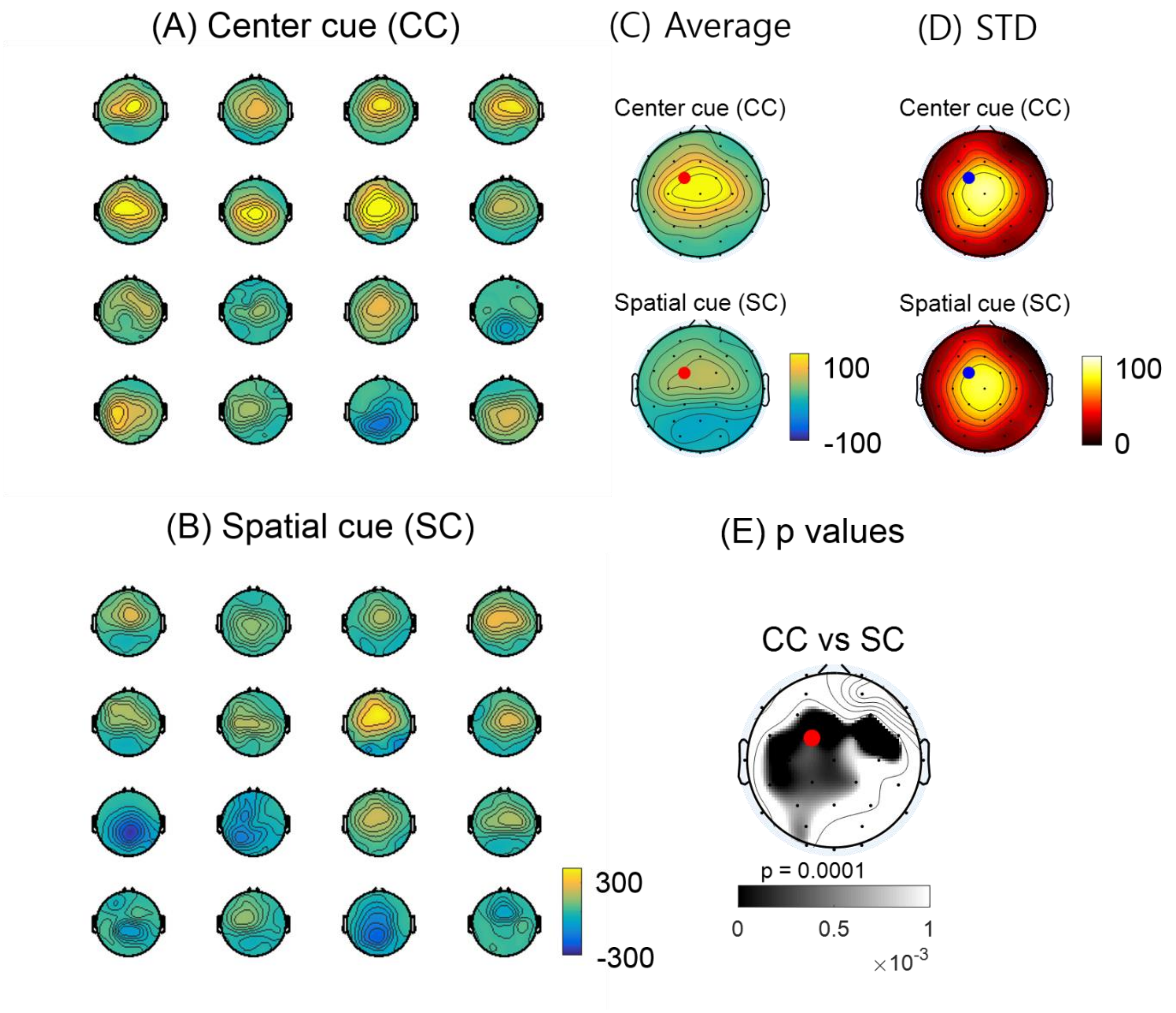

Figure S5 Normalized individual topographies for each subject (supplemental information for Figure 4E). Sixteen topographies of (A) center cue and (B) spatial cue are plotted. (C) Averaged topographies of center cue and spatial cue. (D) Topographies of standard deviation over sixteen subjects. (E) Topography of p values (corrected by FDR). Red or blue marked channel location (FC1) had the smallest p value (FDR corrected p=0.0001).

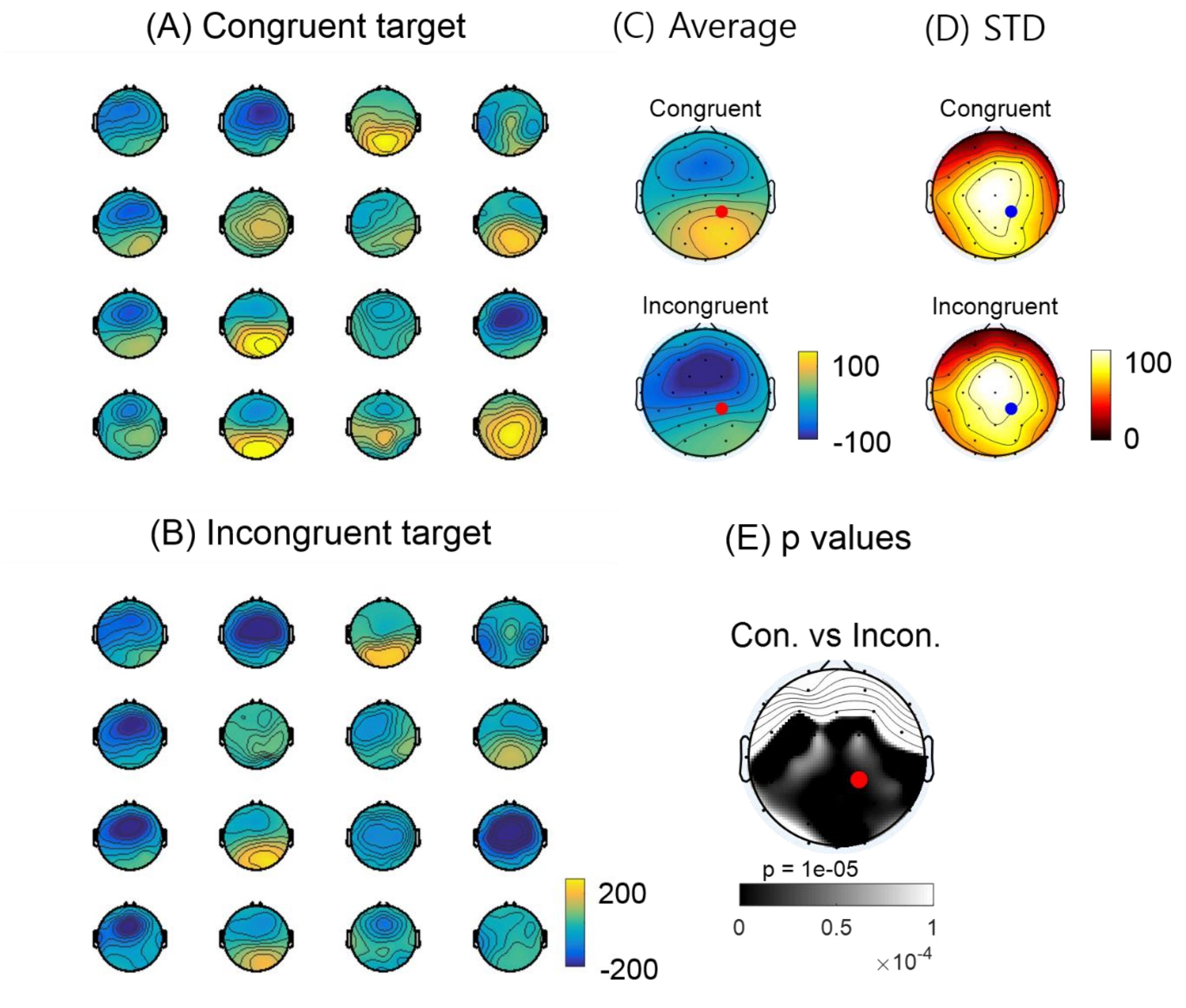

Figure S6 Normalized individual topographies for each subject (supplemental information for Figure 4F). Sixteen topographies of (A) congruent target and (B) incongruent are plotted. (C) Averaged topographies of congruent target and incongruent target. (D) Topographies of standard deviation over sixteen subjects. (E) Topography of p values (corrected by FDR). Red or blue marked channel location (CP2) had the smallest p value (FDR corrected p=0.00001).

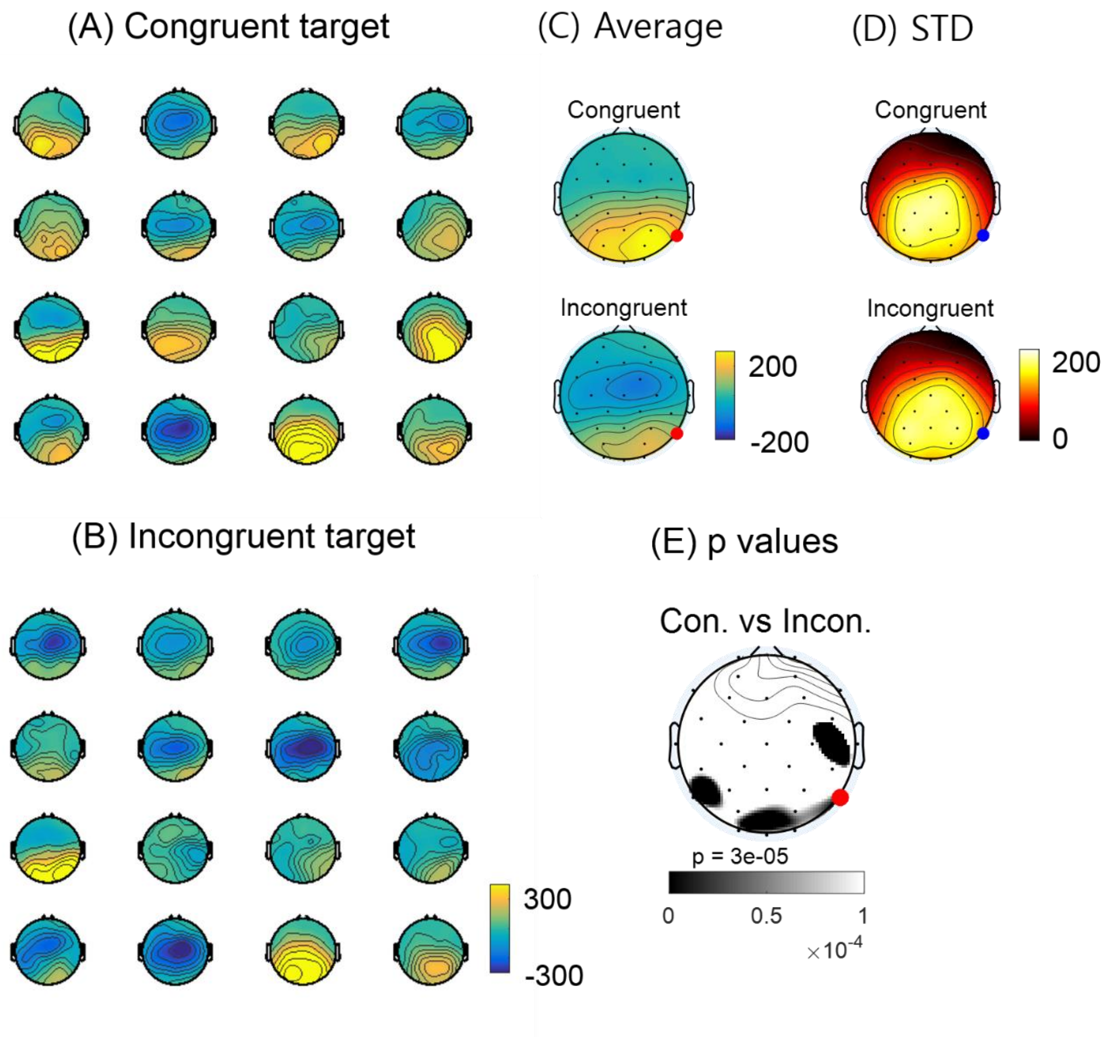

Figure S7 Normalized individual topographies for each subject (supplemental information for Figure 4G). Sixteen topographies of (A) congruent target and (B) incongruent are plotted. (C) Averaged topographies of congruent target and incongruent target. (D) Topographies of standard deviation over sixteen subjects. (E) Topography of p values (corrected by FDR). Red or blue marked channel location (P8) had the smallest p value (FDR corrected p=0.00003).

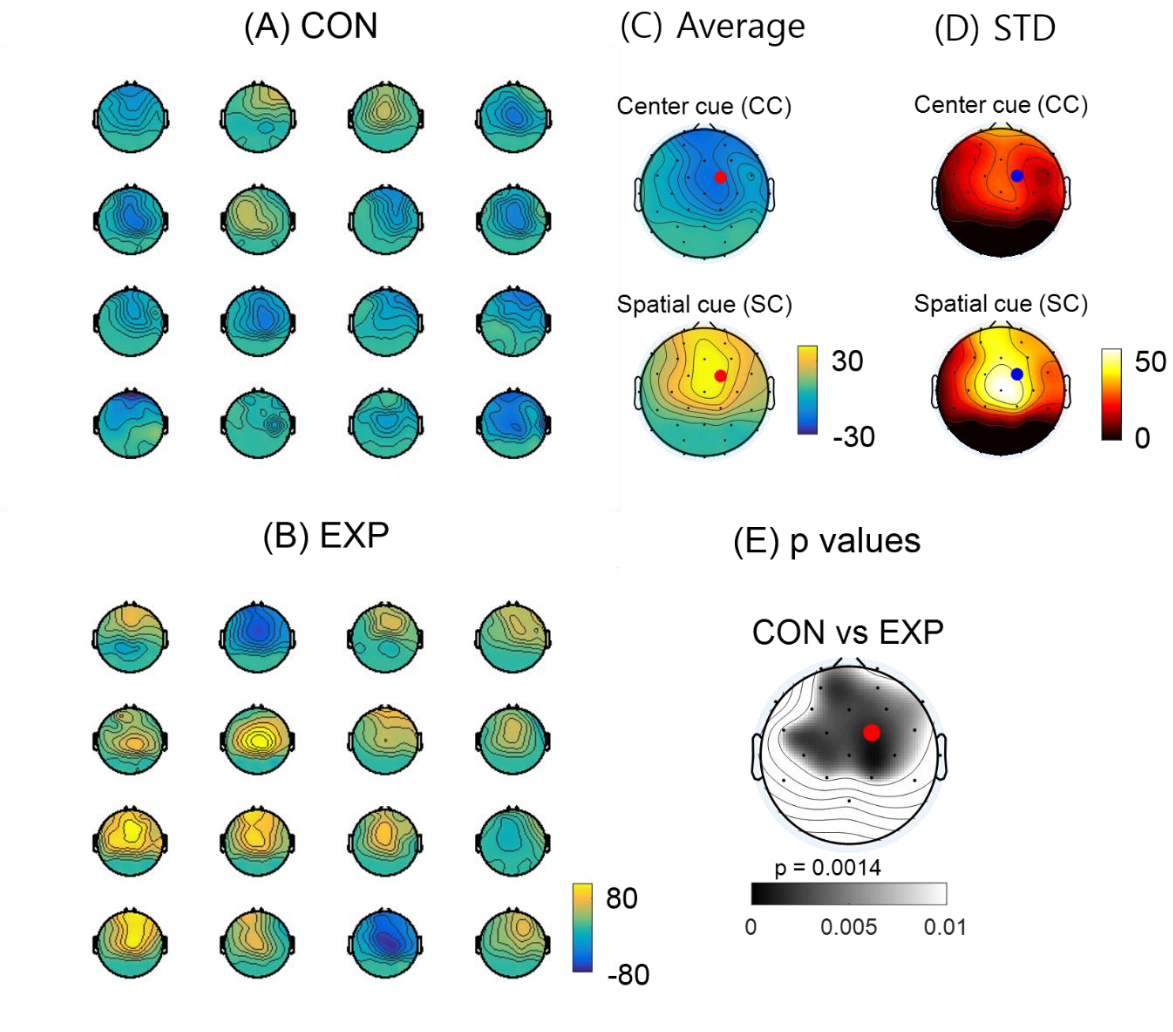

Figure S8 Normalized individual topographies for each subject (supplemental information for Figure 5). Sixteen topographies of (A) control and (B) experimental group are plotted. (C) Averaged topographies of control and experiment group. (D) Topographies of standard deviation over sixteen subjects. (E) Topography of p values (corrected by FDR). Red or blue marked channel location (FC2) had the smallest p value (FDR corrected p=0.0014).

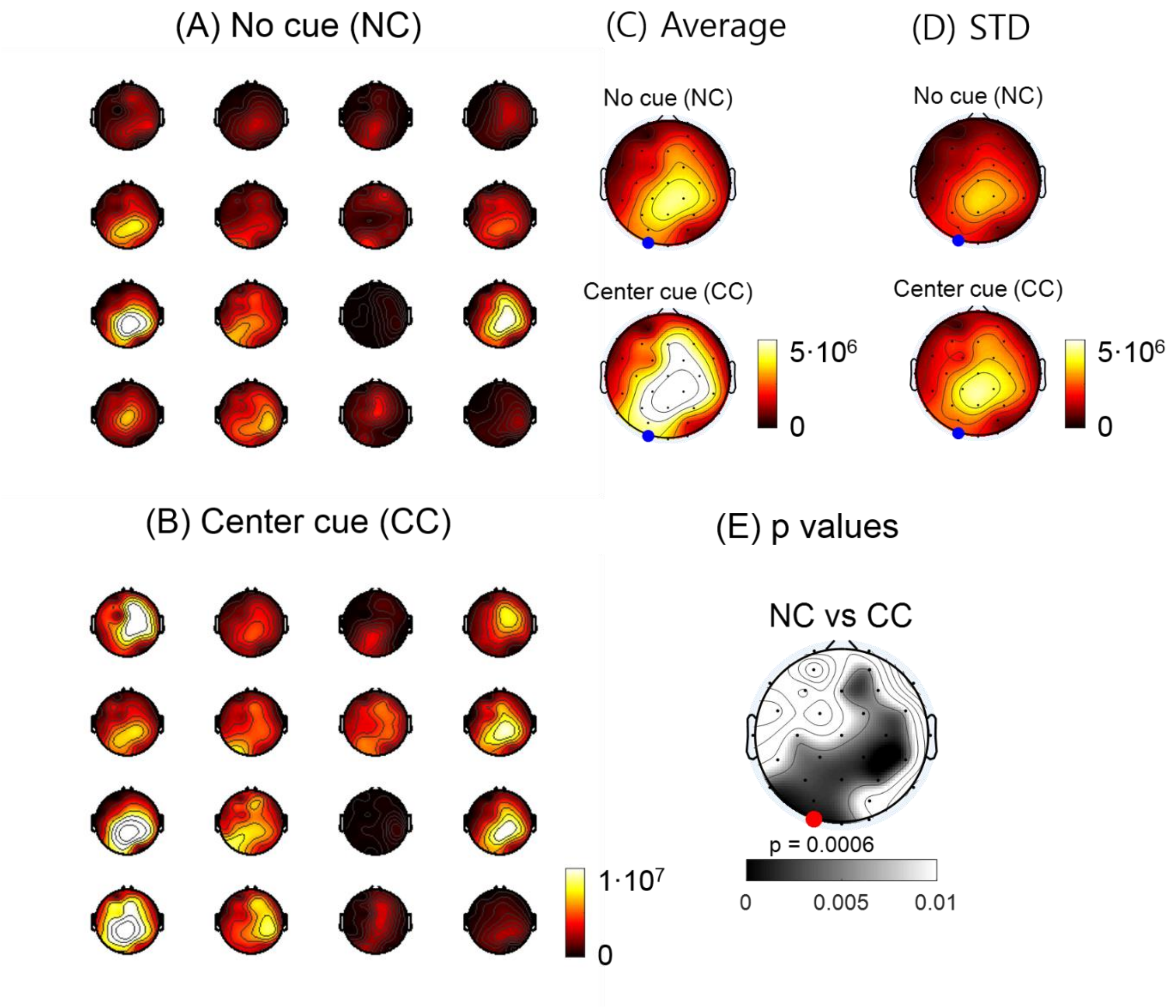

Figure S9 Normalized individual topographies for each subject (supplemental information for Figure 6A). Sixteen topographies of (A) no cue and (B) center cue are plotted. (C) Averaged topographies of no cue and center cue. (D) Topographies of standard deviation of no cue and center cue. (E) Topography of p values (corrected by FDR). Red or blue marked channel location (O1) had the smallest p value (FDR corrected p=0.0006).

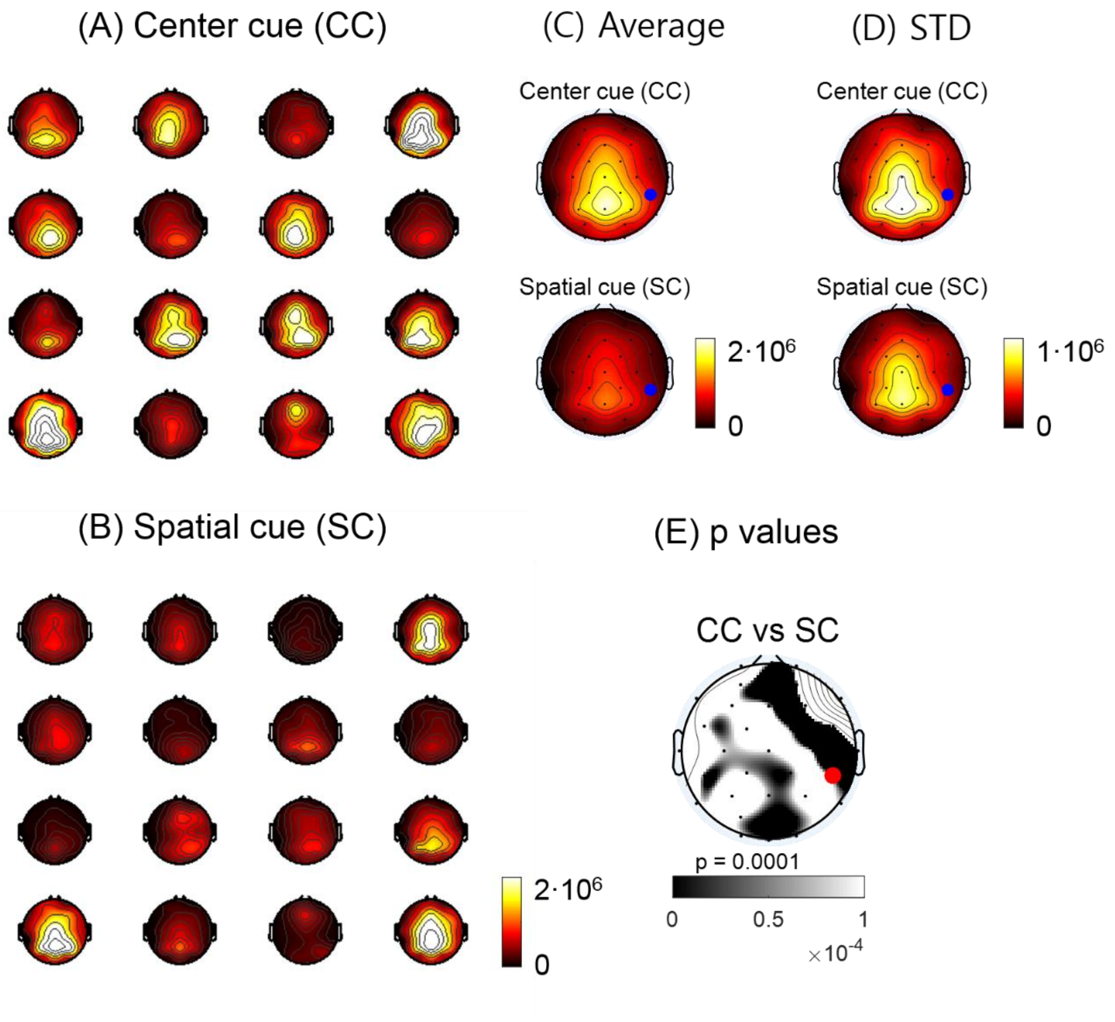

Figure S10 Normalized individual topographies for each subject (supplemental information for Figure 6B). Sixteen topographies of (A) center cue and (B) spatial cue are plotted. (C) Averaged topographies of center cue and spatial cue. (D) Topographies of standard deviation over sixteen subjects. (E) Topography of p values (corrected by FDR). Red or blue marked channel location (CP6) had the smallest p value (FDR corrected p=0.0001).

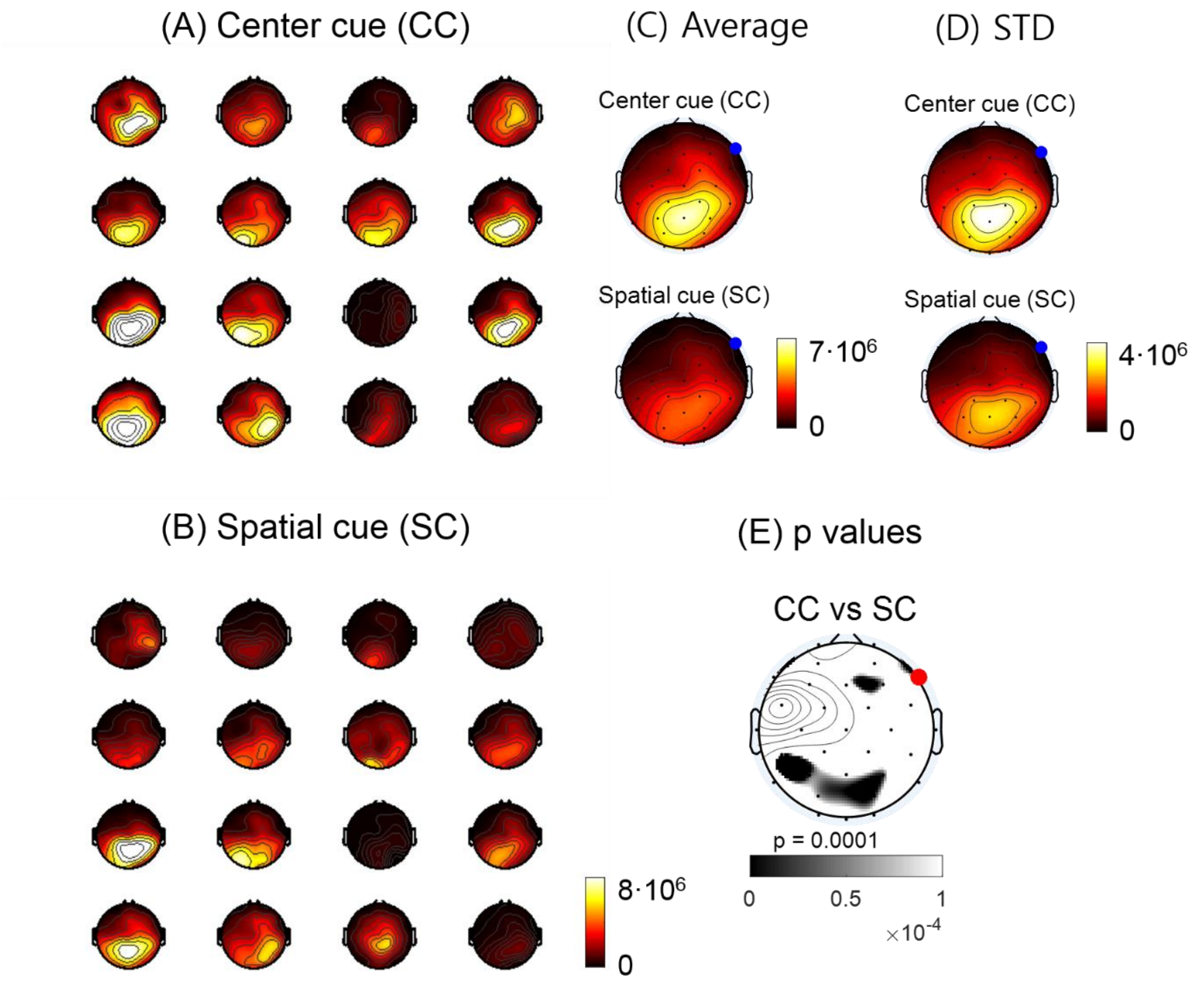

Figure S11 Normalized individual topographies for each subject (supplemental information for Figure 6C). Sixteen topographies of (A) center cue and (B) spatial cue are plotted. (C) Averaged topographies of center cue and spatial cue. (D) Topographies of standard deviation over sixteen subjects. (E) Topography of p values (corrected by FDR). Red or blue marked channel location (F8) had the smallest p value (FDR corrected p=0.0001).

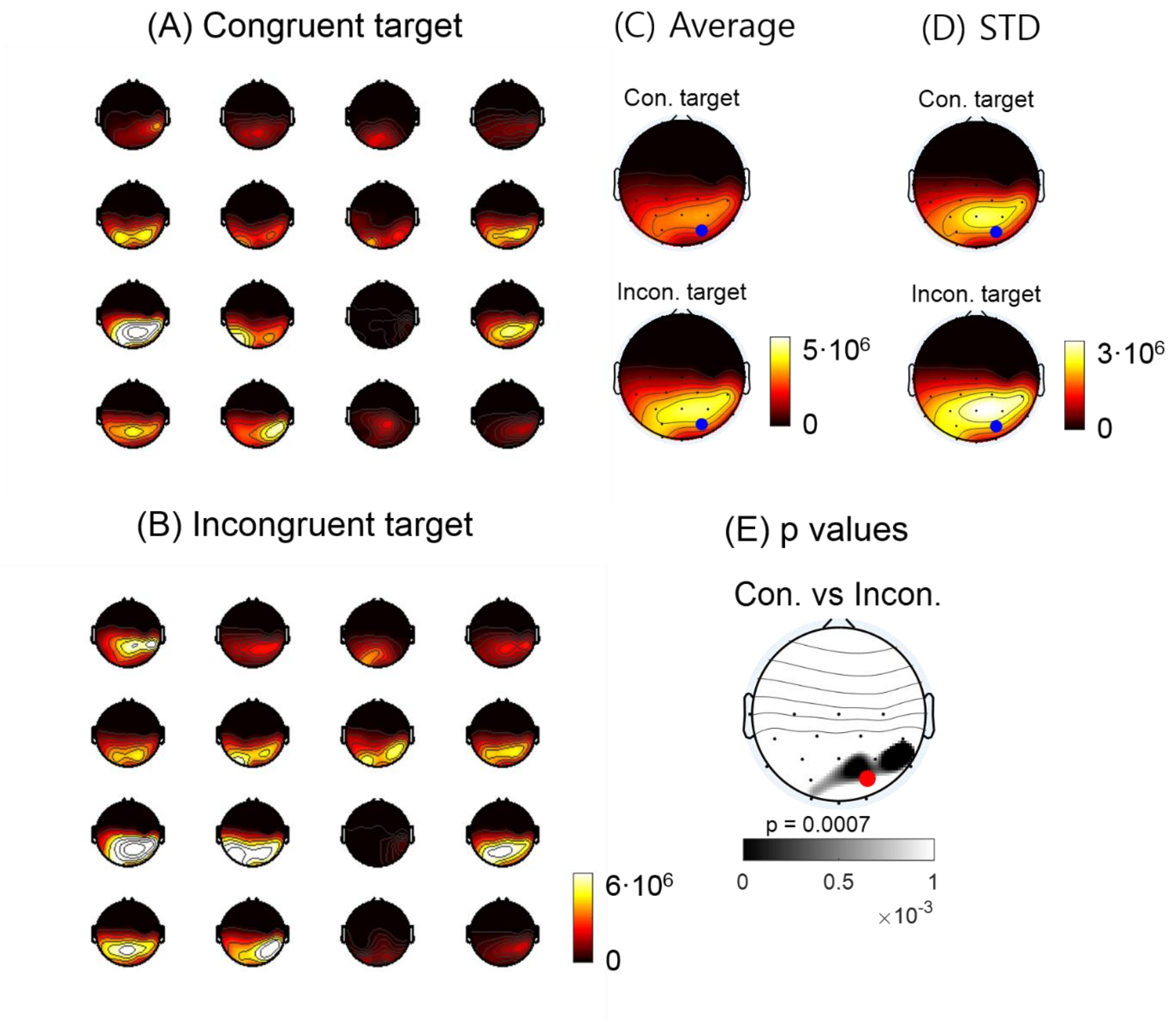

Figure S12 Normalized individual topographies for each subject (supplemental information for Figure 6D). Sixteen topographies of (A) congruent target and (B) incongruent are plotted. (C) Averaged topographies of congruent target and incongruent target. (D) Topographies of standard deviation over sixteen subjects. (E) Topography of p values (corrected by FDR). Red or blue marked channel location (PO4) had the smallest p value (FDR corrected p=0.0007).

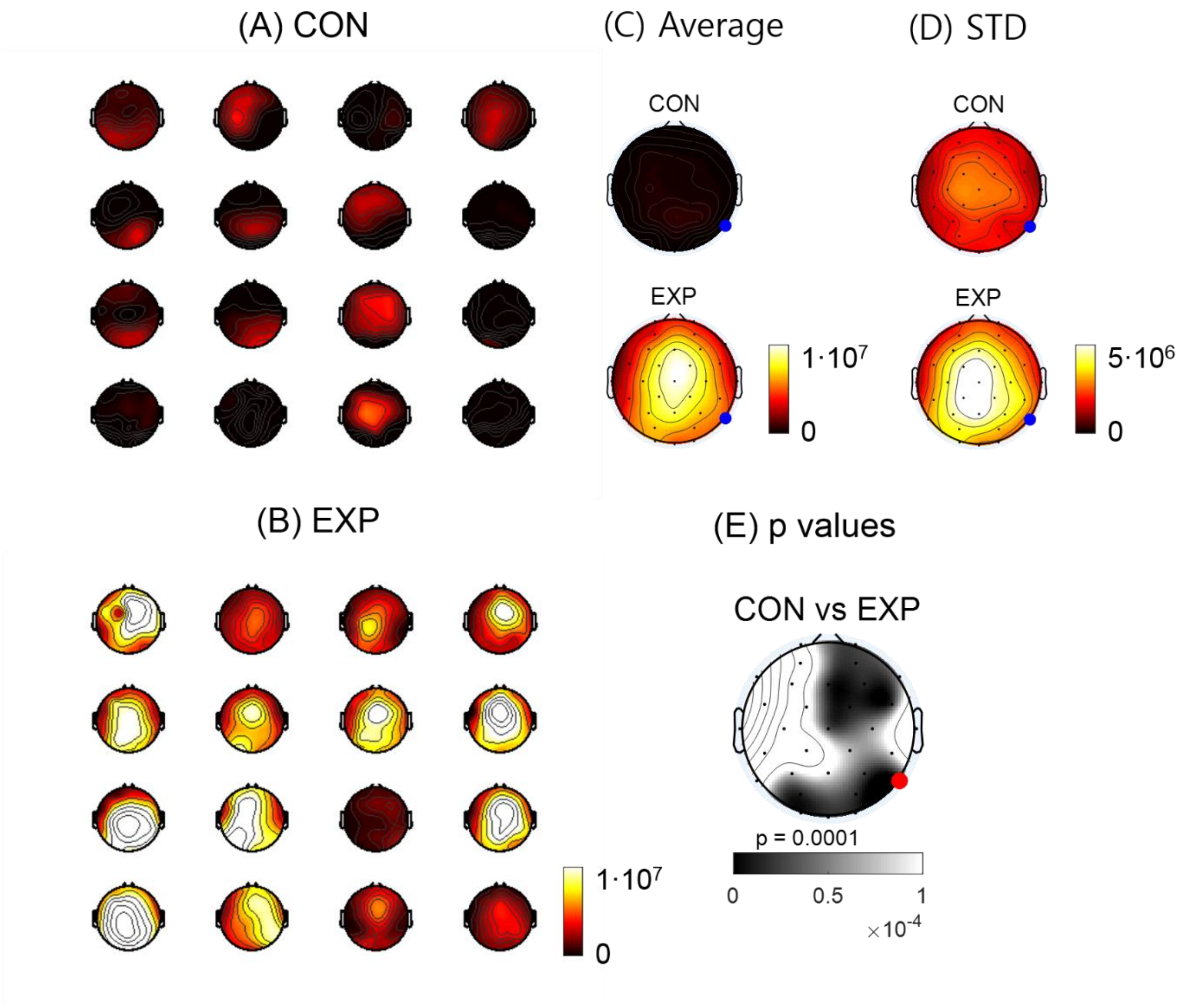

Figure S13 Normalized individual topographies for each subject (supplemental information for Figure 7A). Sixteen topographies of (A) control and (B) experimental group are plotted. (C) Averaged topographies of control and experiment group. (D) Topographies of standard deviation over sixteen subjects. (E) Topography of p values (corrected by FDR). Red or blue marked channel location (P8) had the smallest p value (FDR corrected p=0.0001).

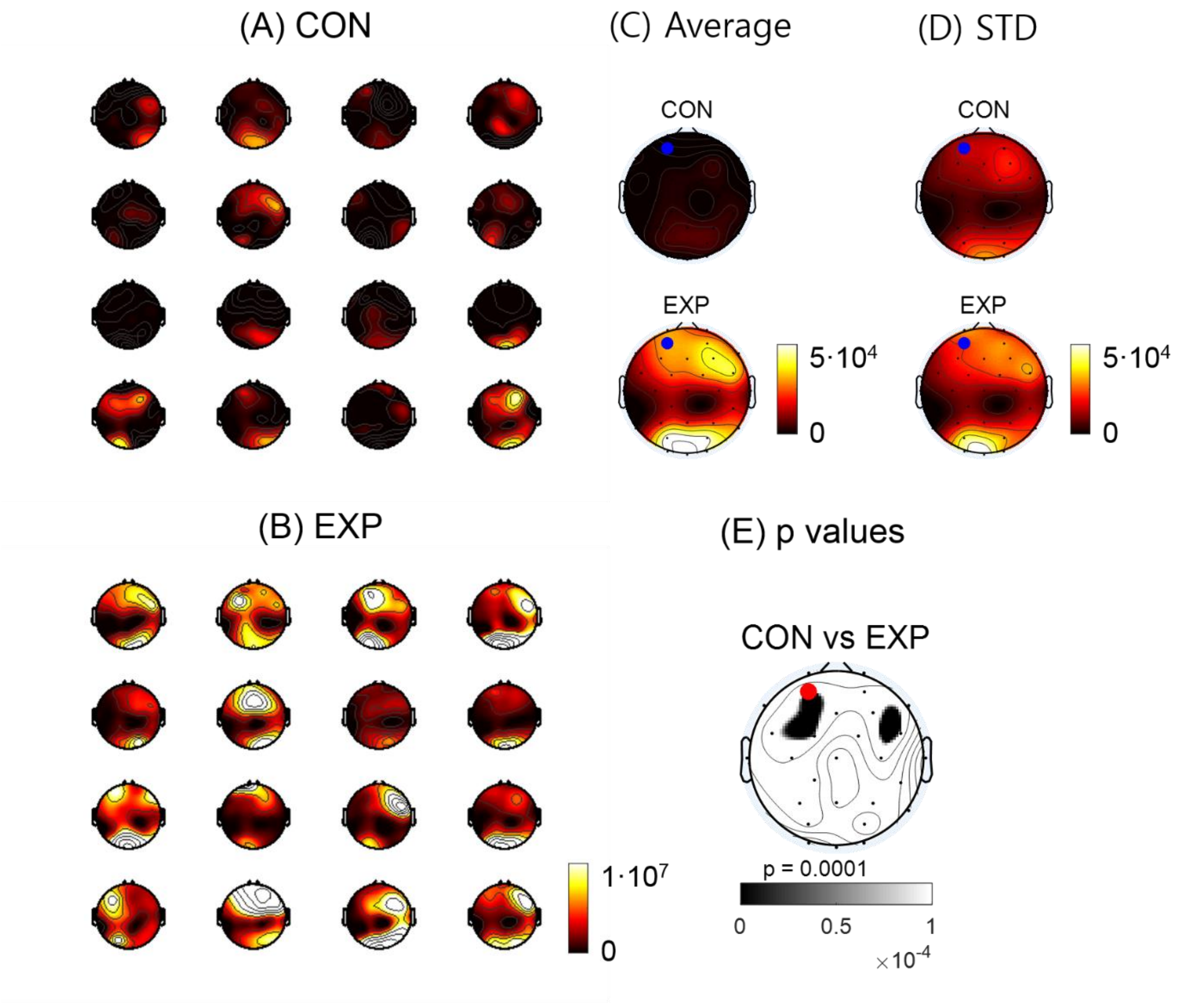

Figure S14 Normalized individual topographies for each subject (supplemental information for Figure 7B). Sixteen topographies of (A) control and (B) experimental group are plotted. (C) Averaged topographies of control and experiment group. (D) Topographies of standard deviation over sixteen subjects. (E) Topography of p values (corrected by FDR). Red or blue marked channel location (AF3) had the smallest p value (FDR corrected p=0.0001).

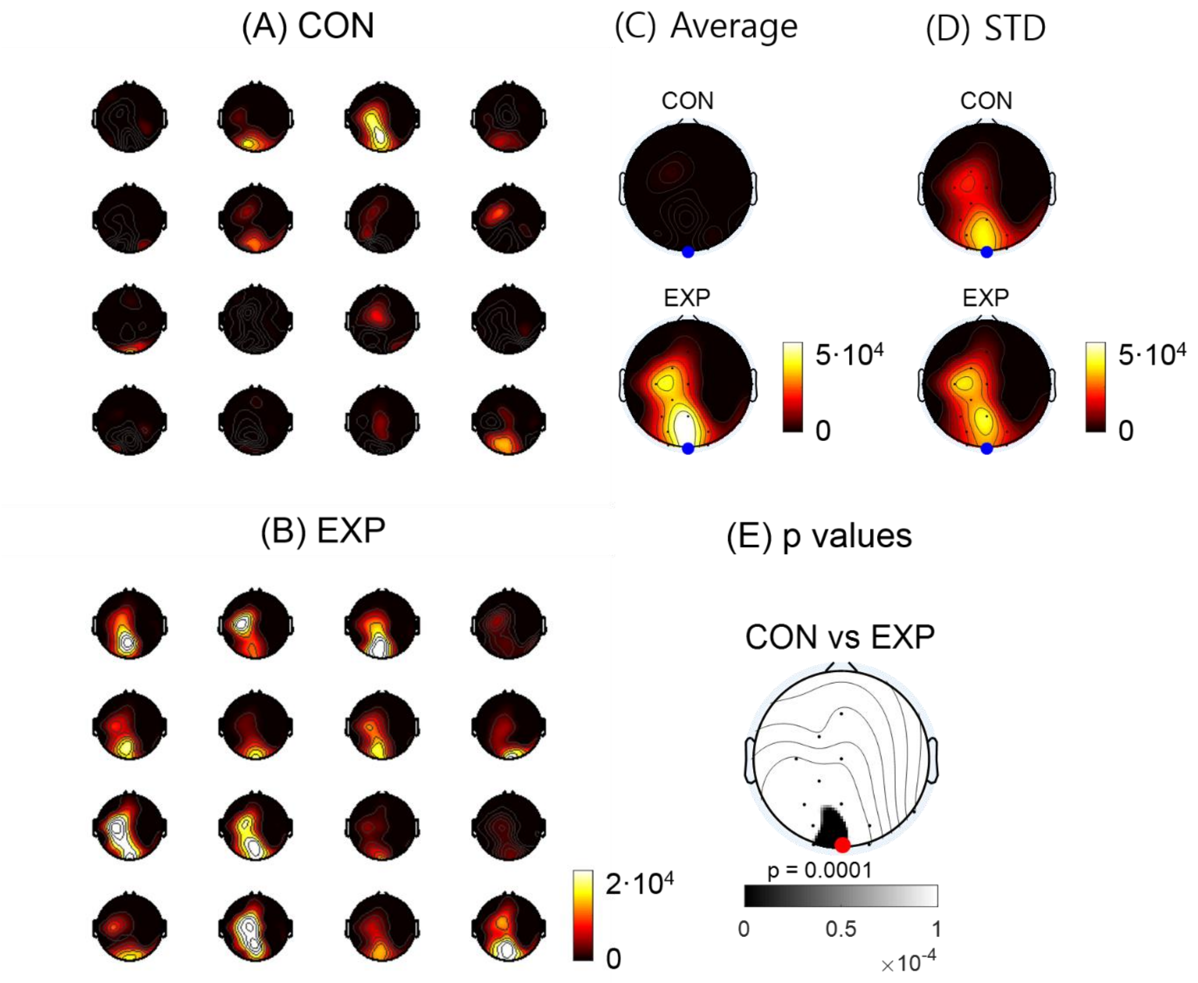

Figure S15 Normalized individual topographies for each subject (supplemental information for Figure 7C). Sixteen topographies of (A) control and (B) experimental group are plotted. (C) Averaged topographies of control and experiment group. (D) Topographies of standard deviation over sixteen subjects. (E) Topography of p values (corrected by FDR). Red or blue marked channel location (Oz) had the smallest p value (FDR corrected p=0.0001).
